# Supplementary figures and images for: Data-driven classification of the certainty of scholarly assertions
Source: PeerJ. 2020 Apr 21;8:e8871. doi: 10.7717/peerj.8871 (PMC7182025; doi:10.7717/peerj.8871)

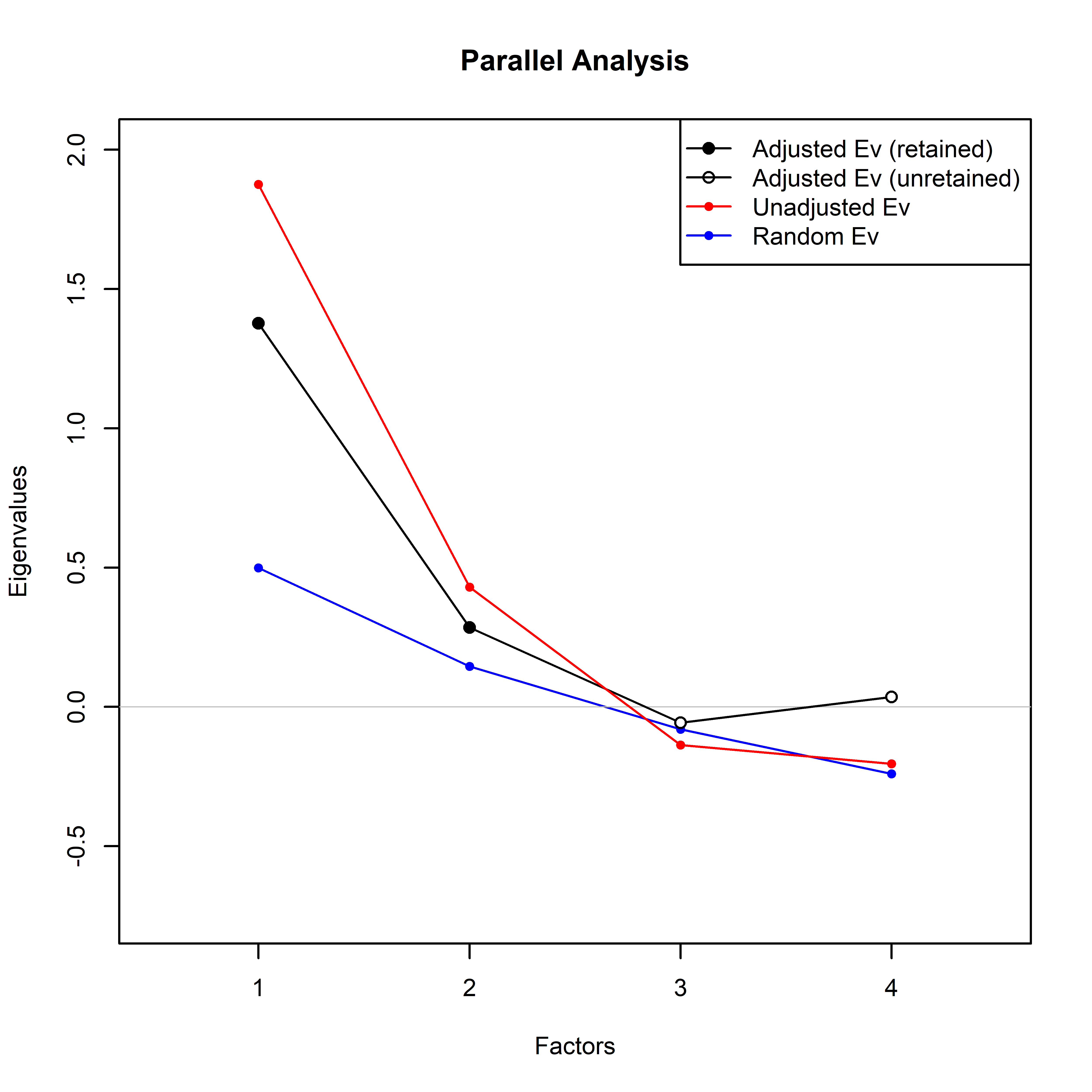

Supplement: Figure S1 — The optimal number of principal components was selected using Horn’s parallel analysis to the certainty categories of the 3 questionnaires. Analysis was carried out using paran function of the R package paran. Runnable code to reproduce these analyses is available in a Jupyter Notebook on Github (https://github.com/Guindillator/Certainty). Fig. S1, S2 and S3 show the result of the Horn’s parallel analysis. [file peerj-08-8871-s001.png]

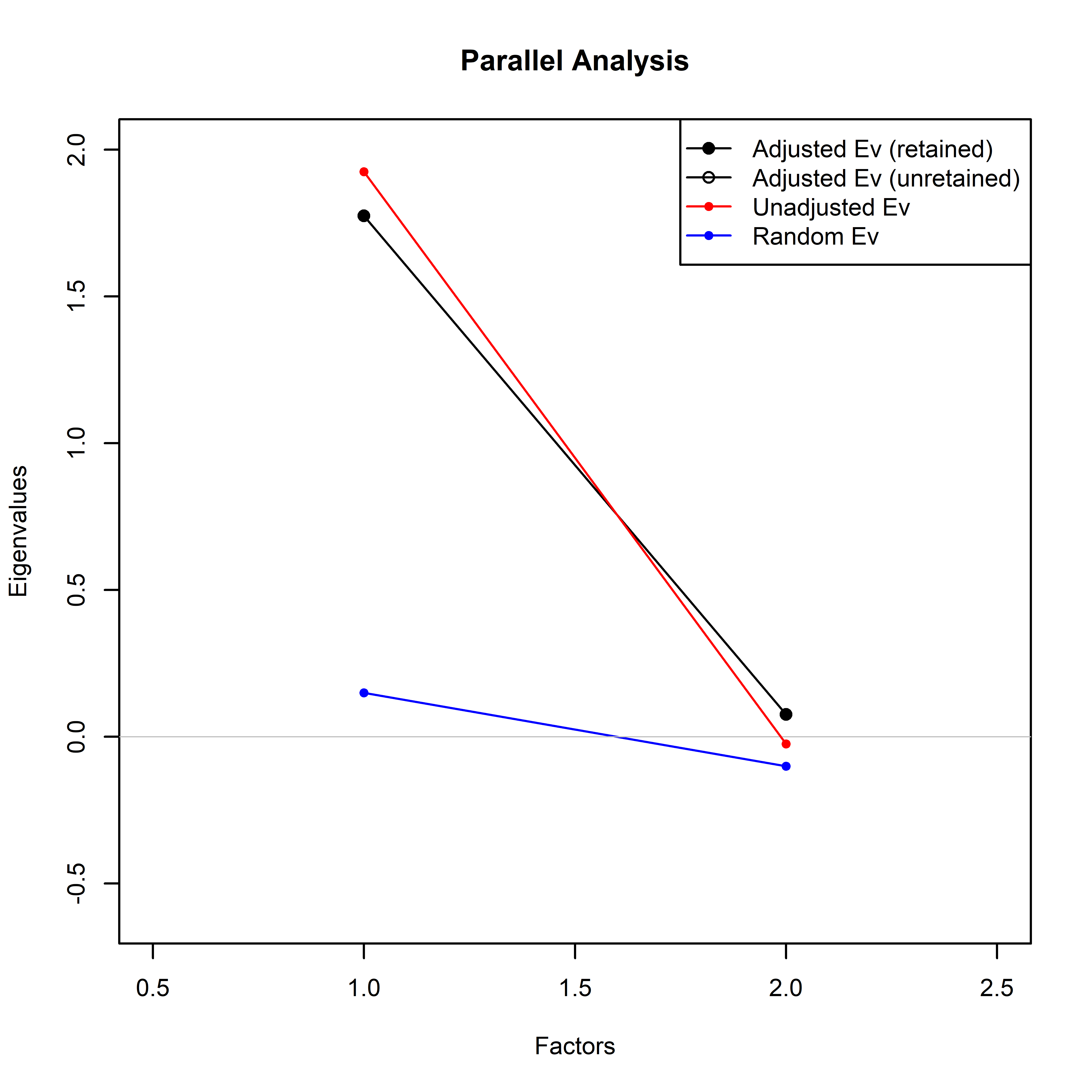

Supplement: Figure S2 — The optimal number of principal components was selected using Horn’s parallel analysis to the certainty categories of the 3 questionnaires. Analysis was carried out using paran function of the R package paran. Runnable code to reproduce these analyses is available in a Jupyter Notebook on Github (https://github.com/Guindillator/Certainty). Fig. S1, S2 and S3 show the result of the Horn’s parallel analysis. [file peerj-08-8871-s002.png]

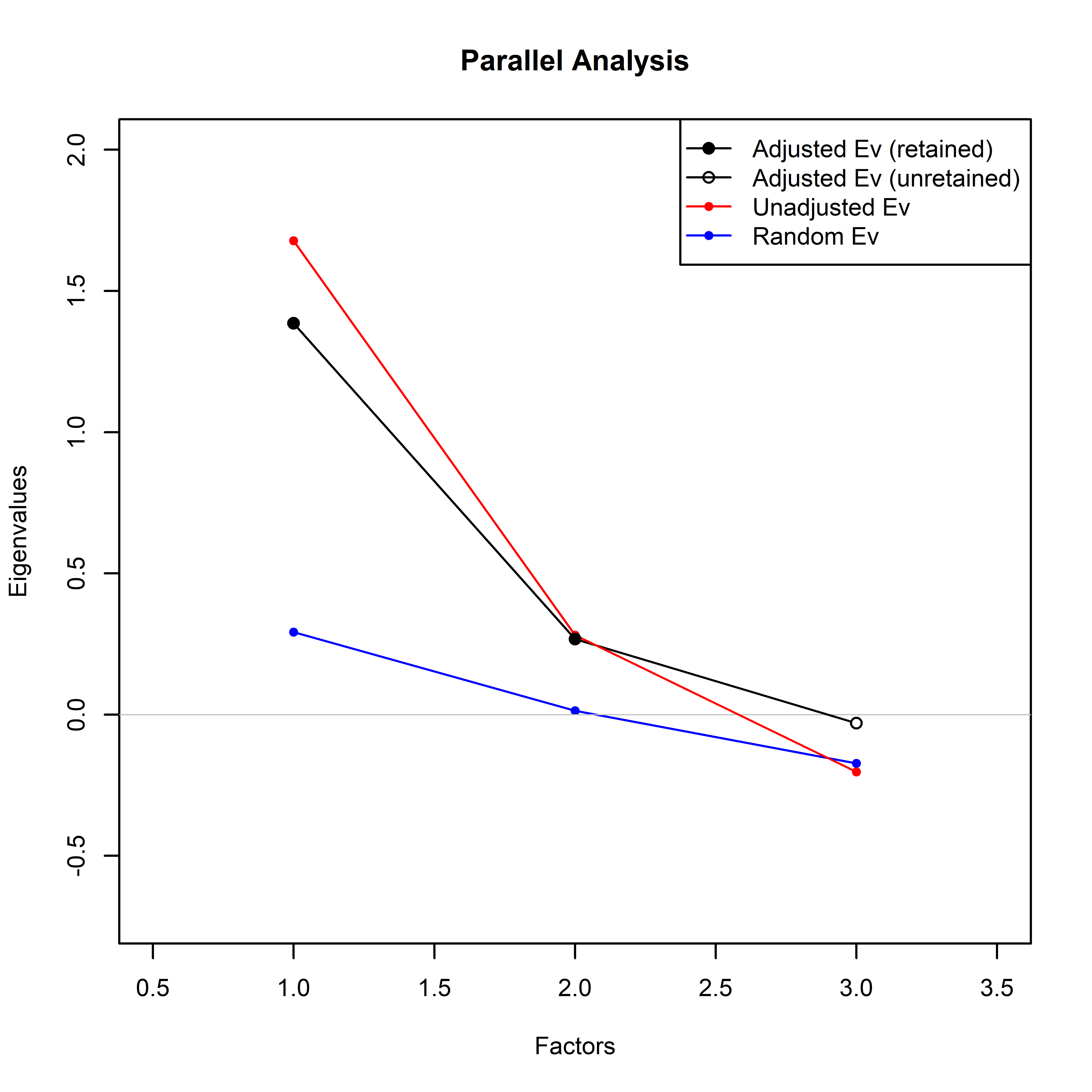

Supplement: Figure S3 — The optimal number of principal components was selected using Horn’s parallel analysis to the certainty categories of the 3 questionnaires. Analysis was carried out using paran function of the R package paran. Runnable code to reproduce these analyses is available in a Jupyter Notebook on Github (https://github.com/Guindillator/Certainty). Fig. S1, S2 and S3 show the result of the Horn’s parallel analysis. [file peerj-08-8871-s003.png]
